# Modelling the range expansion of the Tiger mosquito in a Mediterranean Island accounting for imperfect detection

**DOI:** 10.1186/s12983-017-0217-x

**Published:** 2017-07-27

**Authors:** Giacomo Tavecchia, Miguel-Angel Miranda, David Borrás, Mikel Bengoa, Carlos Barceló, Claudia Paredes-Esquivel, Carl Schwarz

**Affiliations:** 1 Population Ecology Group, IMEDEA (CSIC-UIB), c. Miquel Marqués 21, 07190 Esporles, Spain; 2 Laboratory of Zoology, Department of Biology, University of the Balearic Island, c. Valldemossa s/n, Palma de Mallorca, Spain; 3 Consultoria Moscard Tigre, c. Gremi Passamaners 24, Local 15, 07009 Palma de Mallorca, Spain; 4 Department of Statistics and Acutarian Science, Simon Fraser University, Burnaby, BC Canada

**Keywords:** Tiger mosquito, Site-occupancy model, Population dynamics, Invasion, Range expansion

## Abstract

**Backgrounds:**

*Aedes albopictus* (Diptera; Culicidae) is a highly invasive mosquito species and a competent vector of several arboviral diseases that have spread rapidly throughout the world. Prevalence and patterns of dispersal of the mosquito are of central importance for an effective control of the species. We used site-occupancy models accounting for false negative detections to estimate the prevalence, the turnover, the movement pattern and the growth rate in the number of sites occupied by the mosquito in 17 localities throughout Mallorca Island.

**Results:**

Site-occupancy probability increased from 0.35 in the 2012, year of first reported observation of the species, to 0.89 in 2015. Despite a steady increase in mosquito presence, the extinction probability was generally high indicating a high turnover in the occupied sites. We considered two site-dependent covariates, namely the distance from the point of first observation and the estimated yearly occupancy rate in the neighborhood, as predicted by diffusion models. Results suggested that mosquito distribution during the first year was consistent with what predicted by simple diffusion models, but was not consistent with the diffusion model in subsequent years when it was similar to those expected from leapfrog dispersal events.

**Conclusions:**

Assuming a single initial colonization event, the spread of *Ae. albopictus* in Mallorca followed two distinct phases, an early one consistent with diffusion movements and a second consistent with long distance, ‘leapfrog’, movements. The colonization of the island was fast, with ~90% of the sites estimated to be occupied 3 years after the colonization. The fast spread was likely to have occurred through vectors related to human mobility such as cars or other vehicles. Surveillance and management actions near the introduction point would only be effective during the early steps of the colonization.

## Background

Measuring species range expansion and the pattern of dispersal is a central theme in animal ecology and of particular importance in the management or control of invasive species [39]. Most mathematical models for range expansion assume no false negative for detection of a species, that is to say, if a species is present at a given site, it will always be detected [42]. However, cryptic species or species at the initial phase of the expansion process, might not be detected under a given density threshold [6, 22], which would lead to underestimation of the species prevalence, i.e. number of sites occupied, and the pace of range expansion, i.e. species growth rate. MacKenzie et al. (2006; [26]) proposed an approach based on repeated surveys on sites to estimate the detection probability and the likelihood of species presence accounting for a detection probability <1. In contrast to classical models of range expansion [42], site-occupancy models are discrete in space and time. However, their flexibility permits modelling species occurrence as a function of a continuous spatial or temporal covariates [16] allowing the comparison of predictions of the pattern of colonization similar to those that characterise classical diffusion models. In a simple diffusion model [10] range dynamic is driven only by the intrinsic population growth rate and by the random short-distance movements of individuals [11, 42]. This model predicts that colonization probability co-varies negatively with the distance from the central point or the observed initial site of occupation [27]. In many species, however, random short-distance movements are coupled with long-distance dispersal events, leading to a second type of models characterized by multiple centres of diffusion, an expansion process often referred to as ‘hierarchical diffusion’ or ‘stratified dispersal’ [14, 42]. At a small spatial scale, a negative association between colonization probability and distance from the site of first colonization does not necessarily occur during stratified dispersal because the species can be absent at intermediate distances. However, a diffusion process from each new colonised site would still exist. A third pattern of range expansion is the one resulting from ‘leapfrog’ dispersal movements, with no or little subsequent diffusion [7]. This model predicts high colonization and extinction probability but low diffusion. In this model, the overall occupancy probability would increase as a result of range expansion but with no apparent relationship with the distance from the initial occupied site and without a clear diffusion process.

We used dynamic site-occupancy models [27] to measure the rate of expansion of the Asian Tiger mosquito *Aedes* (*Stegomya*) *albopictus* (Skuse, 1894) (Diptera; Culicidae) in the Island of Mallorca (Balearic Islands, Spain). We contrasted models consistent with different types of range expansion patterns to investigate the underlying dispersal process. The Asian Tiger mosquito is a daytime-active mosquito native to the tropical and subtropical region of southern Asia [13], and is considered to be one of the most invasive species in the world [23]. Its current distribution includes all continents except Antarctica [20]. In Europe, the species was first detected in Albania in 1979 [1], with no records reported in the rest of the continent until 1990s, when it appeared in Italy [19] from where it rapidly spreads to Southern and Central Europe [29, 41]. The first detection in mainland Spain occurred in 2004 in Sant Cugat del Vallés (Catalonia, Spain; [2]). Currently its distribution in Spain includes most of the Mediterranean coast as well Northern areas of the Iberian Peninsula ([8], 2016). In Mallorca (Balearic Islands) the species was first detected in 2012 in 5 municipalities [30] and it rapidly spread to Ibiza in 2014 (Barceló et al., 2015) and Menorca in 2016 [3]. Despite being able to feed upon different hosts depending on their availability [45], *Ae. albopictus* adults obtain blood preferably from humans [32]. The expansion of the Tiger mosquito in Europe has recently created public concerns for its possible role in the transmission of the Zika virus, responsible of microcephaly in newborns of infected mothers (ECDPC, 2016) and as a potential vector for Dengue and Chikungunya viruses [34, 46]. Understanding the spread of invasive mosquito species would thus provide important information needed for a successful control and prevention campaigns. At a large spatial scale, the presence of *Ae. Albopictus* is associated with the level of rainfall and day time surface temperature and its dispersal is facilitated by human activities [44]. However, pattern of dispersal and distribution at small spatial scale and from the early steps of the colonization process are largely unknown. The isolated character of recently colonized Balearic Islands offers the unique opportunity to follow the invasion process and to determine the mechanisms underlying species diffusion.

Our first aim was to estimate the prevalence of the occupation, i.e. the proportion of sites occupied, and the annual rate of spread, i.e. the proportional change in the number of sites occupied per year. We subsequently investigated the expansion processes by modelling the colonization probability as a linear function of the distance from the site of first reported occupancy. If the expansion followed a diffusion process, we expected the probability of occupancy to abate with the distance from the first reported occupied site. Indeed, diffusion is a slow process for *Ae albopictus* [28] even when compared with other *Aedes* species [12]. Also, under random short-distance dispersal, models in which the probability of occupancy at a given site is a function of the occupancy of the neighbourhood would provide a good description of the data [4, 47]. Alternatively, if range expansion occurred mainly by leapfrog dispersal, we expected neither the distance from the initial points nor models depending on neighbourhood covariates to be adequate.

## Methods

### Mosquito prevalence and occupancy rate

**Fig. 1:**
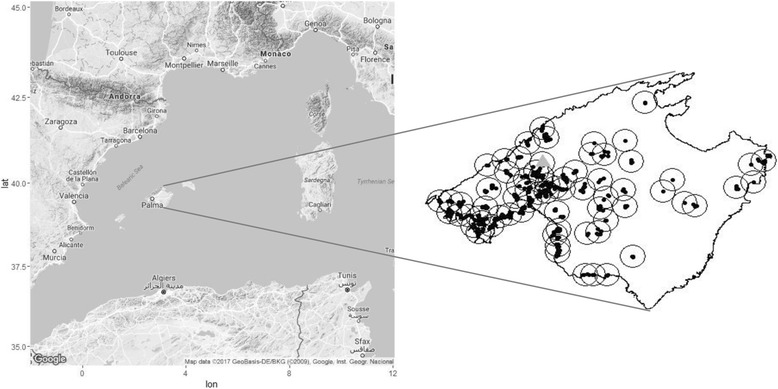
The Island of Mallorca with the location of the 70 sites (*black dots*) monitored for the presence of *Ae. Albopictus*. The *circles* indicate an area of diameter equal to the average nearest neighbor distance between the sites (3.6 Km). The *grey triangle* is the site of first observation in 2012. Note that the more eastern locations have been monitored in 2012 only when the species was first reported in Mallorca

Mallorca Island is the largest and most populated island of the Balearic archipelago, Eastern Spain, with a surface of 3640 km^2^ and about 860 × 10^3^ inhabitants (in 2015). Since the first record of *Ae. albopictus* in Mallorca in 2012, a network of 784 oviposition traps (described in [30]) was deployed in 40 municipalities to monitor the species distribution and range expansion. The monitoring scheme changed over the years resulting in data sparseness. As a consequence, we first restricted the analysis to data collected during the 3 months of maximum abundance of *Ae. Albopictus* (September–November) during the period 2012–2015. To further reduce data sparseness, we used a cluster-by-distance analysis to group neighboring traps into 70 clusters (‘sites’, hereafter; Fig. 1). The clustering distance threshold was arbitrarily chosen as a good compromise between data richness and number of sites monitored. Besides reducing data sparseness, the clustering allowed a more straightforward interpretation of the yearly occupancy rate because the number and the identity of clusters remained roughly constant throughout the study (Table 1). Nevertheless the dataset was unbalanced and information gaps persisted for some locations (e.g. 23 locations have been sampled in only 1 year). For each site in the dataset we recorded the distance from the location of first observation in the municipality of Bunyola, about 15 Km north the main city of Palma. Clustering and distance analyses were conducted using program R v3.3.1 [38].

**Table 1:** Presence-absence data of tiger mosquitoes from 70 unique sites monitored during autumn 2012 to 2015

Year	Number of unique sites monitored	Observed occupancy rate (%)	Maximum distance from first reported observation (km)
2012	38	26	21.3
2013	39	26	30.1
2014	44	57	31.6
2015	44	93	41.5

### Modelling site-occupancy accounting for detection probability

The occupancy dynamic at each site was investigated using dynamic site-occupancy models [26, 18] in which the occurrence of mosquitoes at site *i* on a given time *t*, z_i,t_, is considered as a latent state governed by the occupancy probability, ψ_t_. Changes in the occupancy over time can be described as in metapopulation dynamics by the extinction, ε, and colonization, γ, probabilities. Hence the initial occupancy state, at time 1, is assumed to be

$$ {z}_{\mathrm{i},1}\sim \mathrm{Bernoulli}\left({\Psi}_1\right) $$

whereas in subsequent period is:

$$ {\mathrm{z}}_{\mathrm{i},\mathrm{t}} \mid {\mathrm{z}}_{\mathrm{i},\mathrm{t}\hbox{-} 1}\sim \mathrm{Bernoulli}\left({\mathrm{z}}_{\mathrm{i},\mathrm{t}\hbox{-} 1}\left[1\hbox{-} {\upvarepsilon}_{\mathrm{t}\hbox{-} 1}\left]+\right[1\hbox{-} {\mathrm{z}}_{\mathrm{i},\mathrm{t}\hbox{-} 1}\right]{\upgamma}_{\mathrm{i},\mathrm{t}\hbox{-} 1}\right) $$

The actual observations, y_i,t_, on a site *i* at time *t* are treated as conditional on the occurrence probability and the probability, p_t._, to detect the species when present as:

$$ {\mathrm{y}}_{\mathrm{i},\mathrm{t}} \mid {\mathrm{z}}_{\mathrm{i},\mathrm{t}}\sim \mathrm{Bernoulli}\left({\mathrm{z}}_{\mathrm{i},\mathrm{t}}{\mathrm{p}}_{\mathrm{t}}\right) $$

By combining the yearly estimates of γ and ε it is possible to calculate several derived quantities such as i) the probability of occupancy in any given year, ψ_t_ = ψ_t-1_(1-ε_t-1_) + (1-ψ_t-1_) γ_t-1_ and ii) the proportional increase in the probability of occupancy λ_t_ = ψ_t+1_ / ψ_t_ [25]. The proportion of sites occupied at equilibrium, ψ_eq_, that leads to ψ_t_ = ψ_t+1_, can be calculated using the average colonization and extinction probabilities as ψ_eq_ = γ / (γ + ε). In an increasing population typically ψ_1_ < ψ_eq_ while ψ_1_ > ψ_eq_ when population is decreasing [16, 37].

### Modelling the pattern of expansion through site-occupancy models

The presence of *A. albopictus* at each site was first modelled by assuming the initial occupancy, ψ_1_, the extinction, ε, colonization, γ, and detection, p, probabilities varied over time (years), denoted by the model ψ(t)γ(t)ε(t)p(t). This general model was used to estimate the occupancy rate, the turnover, the occupancy growth rate and the extinction probability over time. The model ψ(t)γ(t)ε(t)p(t) does not assume any diffusion process and it is consistent with a leapfrog dispersal pattern in which colonization and extinction change over time but without a particular spatial pattern. We then considered a set of models assuming that the observed mosquito range resulted from a diffusion process. We first modelled the initial occurrence, ψ_1_, and the colonization, γ, probabilities as:

1 $$ \mathrm{logit}\left({\theta}_{i,t}\right)={\alpha}_t+{\beta}_t{X}_{i,t} $$

where *θ* refers to the probabilities ψ_1_ or γ and *X* to the distance (standardized) from the site of fist observation (noted ‘*dist*’ in model notation). This approach was used by MacKenzie et al. (2006) to model the expansion of the House finch *Carpodacus mexicanus* (Müller) in North America. Although the model would be consistent with a diffusion process, it does not include the mechanism itself [47]. Following [47], we constrained the colonization probability, γ, at a given site, *i,* to be dependent on the yearly occupancy rate, ${\overset{-}{\psi}}_t$ *,* within the site neighbourhood, as logit(*γ*_*i,t*_) = *α*_*t*_ *+ β*_*t*_ $\overset{-}{\psi_t}$. The autocovariate *ψ*_*t*_ is:

2 $$ {{\overset{-}{\psi}}_t}^n=\frac{1}{l}{\sum}_{j\varepsilon \left\{{n}_i\right\}}{\psi}_{j,t} $$

and it is defined as the probability of occupancy of the neighbouring patch *j,* and *l* is the number of patches located in the neighbourhood. The neighbourhood can be made by the adjacent patches [4] or by the total patches in the study area as an average measure of the overall occupancy rate [47]. Although at different scales, both models are consistent with either a gradual range expansion through diffusion or a stratified diffusion processes. In theory, if all adjacent cells contribute equally, the two models would only differ in the definition of the neighborhood. In our case, however, this similarity does not hold because the sites monitored were unequally spaced and a further clustering was necessary to define the adjacent sites. To do this, we considered a grid made of 4 × 4 km cells (*n* = 263), a rounded measure of the average nearest neighbour distance between sites. In the majority of the 70 sites monitored (*n* = 56), there was a single site per cell, while 7 cells contained two sites. In this respect the two autoregressive functions cannot be compared because they refer to a different number of sites.

We used a Bayesian framework to estimate model parameters [17]. Bayesian analyses were conducted in WinBUGS [24] using uninformative priors for model parameters (uniform distribution from −20 to +20 for linear predictors and 0 to 1 for probabilities). The posterior distributions of parameters were sampled, using 3 chains and 25,000 simulations (the first 5000 discarded as a burnin period). Model selection in Bayesian analyses is not straightforward [43]. Across nested models, selection can be done using the Deviance Information Criterion (DIC), a generalisation of the Akaike’s Information Criterion (AIC; [5]) for hierarchical models. However, comparisons between models with and without an autoregressive structure cannot be performed using the DIC because the DIC is computed at different levels in the hierarchy of data and parameters. Model adequacy was thus assessed by inspecting the estimates and their standard deviations. We report the DIC of all models, but we warn readers that this should not be taken as a strict criterion for model explanatory power.

## Results

### Mosquito prevalence and occupancy rate

**Fig. 2:**
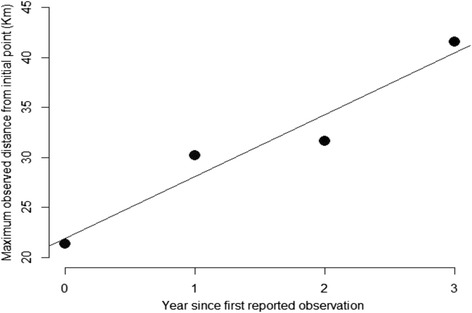
Maximum distance of a observed occurrence from the point of first observation by year. The *solid line* indicates the expected values assuming a linear diffusion. The speed of the observed expansion is 6.2 km per year (slope of the regression line)

The observed edge of the mosquito distribution, i.e. the site at the greatest distance from the initial reported occupancy, was expanding during the study period at an average rate of 6.1 km per year (Fig. 2). Observed site occupancy rate assuming a detection probability of 1.00 were 0.26 (*n* = 38), 0.26 (*n* = 39), 0.57 (*n* = 44) and 0.93 (*n* = 44) in 2012, 2013, 2014 and 2015, respectively (Table 1). However, the site-occupancy model, ψ(t)γ(t)ε(t)p(t), assuming all parameters time-dependent revealed that the detection probability varied from 0.23 in 2013 to 0.85 in 2015, being less than 1 in all years (Table 2). As expected, this model led to estimated occupancy probabilities higher than those observed (0.35, 0.59, 0.57 and 0.90 in 2012, 2013, 2014 and 2015, respectively; Table 2). The probability of local extinction was generally high (average 0.38), but it dropped to 0.07 in 2015 (Table 2). The initial occupancy rate in 2012 (ψ_2012_ = 0.351) was lower than the expected occupancy rate at equilibrium (ψ_eq_ = 0.661) confirming the observed range expansion over the study area. The average growth rate in the occupancy probability was 1.50, equivalent to a 50% increase per year in the number of sites occupied by the species per year. However, this rate of increase was not constant and the range expansion greatly increased after the initial colonization and during the last year (periods 2012–2013 and 2014–2015, Table 2).

**Table 2:** Modelling the occupancy dynamics of the tiger mosquito in Mallorca Island. ψ = occupancy probability, γ = colonization probability, ε = extinction probability. Effects: t = time effect, dist = distance from the site of first observation, m.ψ = autocovariate based on the average occupancy rate in the whole area, D = autocovariate based on the adjacent occupancy rate (see details in ‘Methods’). Note that ψ(covariate) refers to occupancy rate in 2012 only, the occupancy probabilities for the subsequent years are calculated as derived parameters (see text for details)

Model	Type of dispersal	Autocovariate	Notation	DIC	Reference
1	Initial diffusion + leapfrog	Νο	ψ(dist)γ(t)ε(t)p(t)	352.29	[26]
2	Leapfrog	Νο	ψ(t)γ(t)ε(t)p(t)	353.89	[26]
3	Diffusion	No	ψ(dist)γ(dist)ε(t)p(t)	362.03	[26]
4	Diffusion	Νο	ψ(t)γ(dist)ε(t)p(t)	363.84	[26]
5	Leapfrog / Diffusion	Yes	ψ(t)γ(m. ψ)ε(t)p(t)	374.32	[47]
6	Stratified Diffusion	Yes	ψ(t)γ(D)ε(t)p(t)	406.18	[4]

### Modelling the pattern of expansion through site-occupancy model

**Fig. 3:**
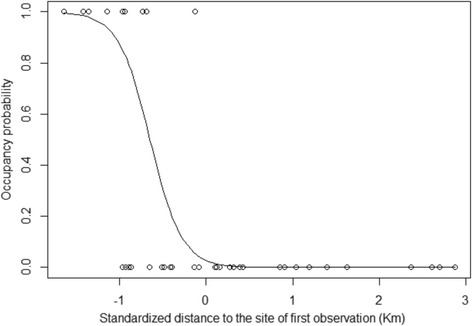
Predicted (*solid line*) and observed (*circle*) site occupancy probability in relation to the distance from the first reported observation (estimates from model assuming an initial diffusion followed by leapfrog movements, noted ψ(dist)γ(t)ε(t)p(t))

**Fig. 4:**
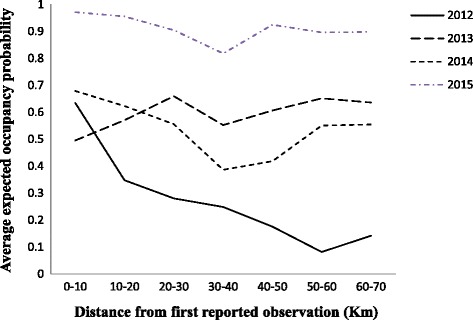
Average site occupancy probability in relation to the distance from the first reported observation (estimates from the model in which all parameters were time-dependent, noted ψ(t)γ(t)ε(t)p(t))

**Fig. 5:**
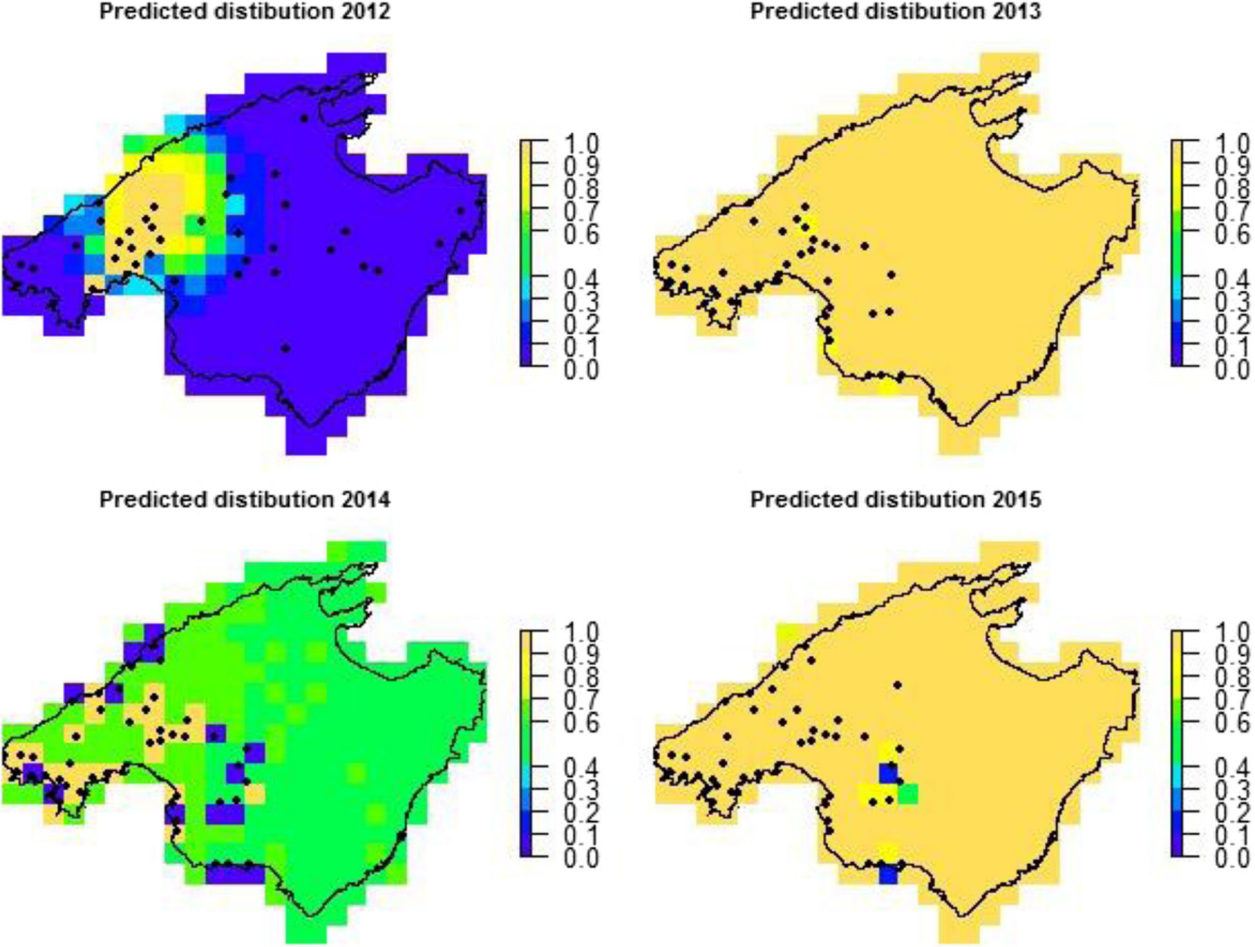
Predicted site occupancy probabilities according to the autoregressive model as in eq. 3 assuming an effect of the neighboring sites (see text for details). *Black dots* are the monitored sites. Note that areas far away from a monitored site display the average estimate of site occupancy. The estimated average occupancy probability by a non-autoregressive model was 0.35, 0.59, 0.57 and 0.90 in 2012, 2013, 2014 and 2015, respectively (Table 2)

The DIC of the general model (DIC model 2 ψ(t)γ(t)ε(t)p(t) = 353.89) improved slightly when the initial probability of occupancy was model as a function of the distance from the point of first reported observation (DIC model 1 ψ(dist)γ(t)ε(t)p(t) = 352.29; Tab. 3). The estimates of α and β (α = −3.581, *β* = −5.537) with the upper 95% credible intervals of β lower than 0.00 (+95%CI = −1.978) indicate that the probability of occupancy in the first year declined as a function of distance. Estimates showed a sharp decline in the occupancy rate, with no occupancy expected at mid-distance between the first reported observation and the farthest site monitored (c. 23 km; Fig. 3). However, the probability of colonization of empty patches in subsequent years did not depend on the distance from the first reported observation (DIC ψ(dist)γ(dist)ε(t)p(t) = 362.03; Table 3-Fig. 4). Models including an autoregression structure in which colonization probabilities were modelled as a linear function of the average neighbouring occupancy rate in the form α + β_1*t*_ ${{\overset{-}{\psi}}_t}^n$ delivered positive but unrealistic standard deviations for the *β*s parameters (mean ± sd: β_1,*2013*_ = 1.91 ± 11.3, β_1,*2014*_ = 4.637 ± 9.51 and β_1,*2015*_ = −6.667 ± 9.46). Similar imprecise estimates were obtained when only adjacent cells were considered (Fig. 5)**.**

**Table 3:** Estimates from model ψ(t)γ(t)ε(t)p(t), assuming all parameters variable over time. Credible interval (CI) at 2.5% and 97.5% are reported. Parameters: ψ_i_ = occupancy probability at time *i*, γ_i_ = colonization probability, e.g. the probability that an empty site is occupied between *i* and *i + 1*, ε_i_ = extinction probability, e.g. the probability that an occupied size at *i* is not-occupied at *i + 1,*, λ_i_ = the growth rate of occupied size between *i* and *i + 1,* ψ_eq_ = occupation probability at equilibrium (see text for details)

Parameter	Mean	Sd	2.5% quantile	97.5% quantile
ψ_2012_	0.351	0.102	0.184	0.58
ψ_2013_	0.587	0.159	0.291	0.887
ψ_2014_	0.568	0.071	0.428	0.704
ψ_2015_	0.899	0.044	0.800	0.968
γ_2012_	0.667	0.201	0.258	0.980
γ_2013_	0.669	0.182	0.251	0.974
γ_2014_	0.862	0.075	0.686	0.973
ε_2012_	0.566	0.24	0.059	0.935
ε_2013_	0.509	0.133	0.252	0.769
ε_2014_	0.073	0.051	0.007	0.197
p_2012_	0.63	0.118	0.384	0.839
p_2013_	0.26	0.103	0.112	0.510
p_2014_	0.80	0.048	0.693	0.882
p_2015_	0.85	0.033	0.782	0.909
λ_2012_	1.846	0.833	0.685	3.867
λ_2013_	1.057	0.388	0.580	2.047
λ_2014_	1.610	0.220	1.261	2.113
ψ_eq_	0.661	0.066	0.548	0.804

## Discussion

### The expansion of the tiger mosquito

The range expansion of the Tiger mosquito in the island of Mallorca has been rapid, with an estimated occupancy of monitored sites probability that increased from 0.35 in 2012 to nearly 0.90 in 2015 and an average annual growth rate in the occupancy of 1.50. Interestingly, local extinction probability was relatively high (except from 2014 to 2015) suggesting a high turnover in the occupied sites. During the first year, when the number of mosquitos was presumably small, treatments with insecticides by private citizens and local administration might have caused temporary extinction of the species in some monitored sites. At present we ignore the intensity and influence of these actions. Extinctions can also have occurred naturally because newly colonized locations are expected to be occupied by a small number of mosquitos. However, colonization probability was also high, leading to a fast re-colonization of locations from which the species disappeared. Under a simple diffusion model [42], the colonization probability should negatively covary with the distance from the site of first observation (MacKenzie et al. 2006). Our results indicated that the distribution of occupied sites during the first year responded to what predicted from a simple diffusion model with random short dispersal movements. In contrast, from 2013 to 2015 the colonization of new sites did not occur as a diffusion process, at least not at the spatio-temporal scale considered here. The fast range expansion is better described by leapfrog dispersal movements, in which an increasing number of sites are occupied each year, but without a clear relationship with the distance from the initial colonization. Autoregressive models delivered unrealistic standard deviations of parameter estimates indicating that a gradual diffusion process is unlikely to have shaped the current distribution at least at the spatial scale considered here. Collantes et al. 2015 [8] mentioned a possible diffusion process of dispersal of *A. albopictus* from its first detection site in mainland Spain in 2004. However, no analyses were conducted for demonstrating such type of dispersal. Other authors also reported leapfrog dispersal movements at larger spatial scale (>500 km) from the site of first detection in Catalonia to the Valencia region (Bueno-Marí et al., 2013). The same pattern of progressive and gradual invasion since the initial point combined with sporadic “jumps” has been also proposed by Roche et al. [35] on a study of the distribution of *Ae. albopictus* in continental France and Corsica. In comparison to other mosquitos, *Ae. albopictus* show a low dispersal capability [12]. According to Marini et al. [28], for example, the average flight distance of *Ae. albopictus* is 119 m per day. However, passive transportation of eggs, through translocations of used tires [33] and adults mosquitoes in vehicles [32] are probably the mechanisms of leapfrog dispersal movements. Despite our conclusions are drawn on a smaller spatial scale than the one previously considered, they are in agreement with what is known of the colonization pattern of the species. However, they are based on the assumption of a single initial colonization event in 2012. We cannot exclude that subsequent colonization (i.e. independent introductions; see for example [15, 36]) occurred after 2012. At the moment it is unknown whether or where this happened, but multiple introductions from mainland through the local airport or the two main ports of Alcudia and Palma would be consistent with a stratified diffusion as predicted by models with auto-covariates The change in colonization and extinction probability in the last year of the study might partly be due to natural causes. For example the amount of rainfall during the summer 2015 was particularly high and can partially explain the high colonization and recapture probabilities (see below). The short period of the study does not permit to fully investigate a relationship between colonization and rainfall but an important role of weather variables has been found in the oviposition dynamics of *Ae. aegypti* in Northwestern Argentina [9] and in the abundance of *Ae albopictus* in the French Riviera (Tar et al. 2013).

### Site occupancy models and species range expansion

The pattern of range expansion of any given species depends on several characteristic such as landscape heterogeneity [11], interspecific competition ([47], 2014), species life-history traits [22], climatic and human-related factors [36, 40].

It is not surprising that analytical models of range expansion are a necessary oversimplification of the underlying biological processes. They allow, nevertheless, some generalizations and the estimates of important parameters that modulate the range expansion [22]. Here we used dynamic site-occupancy models to estimate occupancy rate and colonization speed of the Tiger mosquito accounting for imperfect detection. In contrast to classical models [14], site-occupancy models are discrete in space and time. However, we constrained parameter variability as a function of a site-dependent covariate to deliver predictions consistent with different patterns of colonization as in classical continuous models, i.e. ‘leapfrog’ versus ‘diffusion’. A clear limitation of our work was the difficulty in finding criteria for model selection. However, the problem of contrasting hierarchical models is not only limited to the present study and it is a topic under study in statistical theory and numerical ecology [43]. Additional problems derived from the fast expansion of the mosquito, the limited number of sites monitored and/or a possible spatially consisted driver of the colonization probability. These factors contribute to reduce the variability in occupancy rate among sites leading to numerical problems in model fitting when estimating the effect of the covariate. Beside these limitations, we showed the potential of site-occupancy models in estimating range expansion parameters [26] and can be very useful in the study of disease prevalence and vector dynamics [21, 31]. For example, Padilla-Torres et al. [31] used site-occupancy models to study the prevalence of *Ae. aegypti* and *Ae. albopictus*. They concluded that routine surveillance based on rapid larval surveys led to a lower prevalence of both species and suggest a combined used of ovitrap-based surveillance with analytical methods based on imperfect detection. Finally, MacKenzie and Nichols [27] treated occupancy as a surrogate of abundance. In our case mosquito abundance is more likely to be reflected in the probability of detection, which can be seen as the probability of a trap being used by a gravid female. This is because the conditional probability to detect mosquito larvae in the oviposition traps given that a female has used the trap is equal to 1.00. This would explain why in 2015, when the probability of recapture was high (0.85), the extinction probability was low (0.07). However, the link between abundance and detection is not straightforward because it would depend on multiple factors that have not been considered here, i.e. the habitat characteristics or the availability of alternative breeding sites. The present work is more descriptive than predictive and future research should incorporate additional site-dependent covariates in the models such as habitat type and site attractiveness. This can be done with static (opposite to ‘dynamic’) single-season occupancy-model. Single season models would not allow investigating the expansion process as we did here, but they will avoid trap-clustering and would permit to model mosquito presence using fine scale habitat covariates to predict future distributions.

## Conclusions

Assuming a single colonization event in 2012, we concluded that the rapid expansion of *Ae. Albopictus* in Mallorca Island occurred in two phases. In a first phase the distribution appeared consistent with a diffusion process. This was rapidly followed by leap-frog dispersal events that resulted in an estimated occupancy probability of 90% 3 years after the colonization. The two distinct phases imply that surveillance and management actions near the introduction point would only be effective during the early steps of the colonization. The lowest extinction probability was recorded in the year with the highest amount of summer rainfall suggesting a role of weather covariates on the paste of the expansion. Dynamic site-occupancy models offer a robust analytical framework for the study of range expansion. They are particularly suitable for the study of cryptic species with high turnover as they permit to frame imperfect detections.

## Abbreviations

AIC: Akaike’s Information Criterion
DIC: Deviance Information Criterion
